# Predictors of Adverse 30-Day Outcomes After Right Coronary Artery ST-Elevation Myocardial Infarction

**DOI:** 10.3390/jcm15072595

**Published:** 2026-03-28

**Authors:** Alexander P. Bate, Kyle B. Franke, Ethan Nguyen, Dominic Sutton, Ross L. Roberts-Thomson, Adam J. Nelson, Jessica A. Marathe, Peter J. Psaltis

**Affiliations:** 1 Department of Cardiology, Central Adelaide Local Health Network, Adelaide 5000, Australia; alexander.bate@sa.gov.au (A.P.B.); kyle.franke@sa.gov.au (K.B.F.); dominic.sutton@sa.gov.au (D.S.); ross.roberts-thomson@sa.gov.au (R.L.R.-T.); adam.nelson@adelaide.edu.au (A.J.N.); 2 College of Health, Adelaide University, Adelaide 5000, Australia; ethanhuynhut.nguyen@sa.gov.au; 3 Vascular Research Centre, Lifelong Health Theme, South Australian Health and Medical Research Institute, Adelaide 5000, Australia

**Keywords:** acute coronary syndrome, angiography, cardiogenic shock, myocardial infarction, right coronary artery, right ventricular function

## Abstract

**Background:** There is limited contemporary evidence on predictors of adverse outcomes in ST-elevation myocardial infarction (STEMI) involving the right coronary artery (RCA). We examined this in a single-centre retrospective cohort study, focusing on 30-day outcomes. **Methods:** Consecutive patients presenting to an Australian tertiary hospital between May 2022 and April 2024 with acute STEMI who underwent primary percutaneous coronary intervention (PCI) or rescue PCI were eligible. Patients were divided into STEMI due to RCA and non-RCA culprit lesions, and their characteristics were compared. The primary outcome was a composite of 30-day all-cause mortality and cardiogenic shock. **Results:** Among 320 included patients, the primary composite outcome was similar between the RCA and non-RCA groups (12% vs. 15%, *p* = 0.44), although 30-day mortality was lower in the RCA-STEMI group (2% vs. 8%, *p* = 0.01). In the RCA-STEMI cohort, right ventricular (RV) longitudinal dysfunction on echocardiogram, defined as a tricuspid annular plane systolic excursion (TAPSE) < 17 mm or RV tissue doppler lateral annular systolic velocity (RV S′) < 10 cm/s (*p* = 0.04), and Thrombolysis in Myocardial Infarction (TIMI) flow < 3 in the RV marginal branch post-PCI (*p* = 0.04) were independently associated with the primary outcome. The latter was also associated with a higher risk of intensive care unit admission for cardiogenic shock (*p* < 0.01) and heart failure requiring inpatient diuresis (*p* = 0.02). **Conclusions:** In patients with RCA-STEMI, compromised RV marginal branch flow post-PCI and impaired RV function were independently associated with the composite primary outcome of 30-day all-cause mortality and cardiogenic shock. These characteristics may assist early identification of at-risk individuals who could benefit from pro-active monitoring and early implementation of therapies for cardiogenic shock.

## 1. Introduction

Approximately 40% of all cases of ST-elevation myocardial infarction (STEMI) are caused by occlusions to the right coronary artery (RCA) [1]. Outcomes of RCA culprit STEMI are highly variable and influenced by the degree of right ventricular (RV) involvement [2,3]. People with STEMI and RV involvement are at higher risk of death, cardiogenic shock, atrioventricular block, and ventricular arrhythmia [4,5,6]. However, existing evidence on the clinical, angiographic, and echocardiographic predictors of these adverse outcomes remains limited. The available data indicate that proximal RCA occlusions are the most common culprit lesions for RV dysfunction [7], and occlusions proximal to the RV marginal branch origin are accompanied by increased rates of cardiogenic shock and 30-day mortality [8]. Despite these established associations, the clinical relevance and management strategies for RV marginal branch occlusion remain poorly defined. Beyond angiographic findings, echocardiographic features that reportedly predict increased mortality are reduced tricuspid annular plane systolic excursion (TAPSE) in patients with cardiogenic shock following STEMI [9,10] and the presence of concomitant left ventricular dysfunction in people with acute RV infarction [9,11].

Although these prior studies provide important information about associations with worse outcomes in people presenting with RCA-territory STEMI, little evidence has been generated in the current era of routine primary percutaneous coronary intervention (PCI) and greater access to advanced haemodynamic supports. The current study therefore aimed to assess the clinical, angiographic and echocardiographic predictors of cardiogenic shock and mortality in patients who presented with an RCA culprit vessel STEMI compared with a non-RCA culprit cohort.

## 2. Materials and Methods

This was a single-centre, retrospective cohort study performed at the Royal Adelaide Hospital, South Australia. Approval for waiver of consent for retrospective collection of clinical and angiographic data was approved by the Central Adelaide Local Health Network Human Research Ethics Committee (HREC/25/CALHN/20668).

### 2.1. Study Population

**Figure 1:**
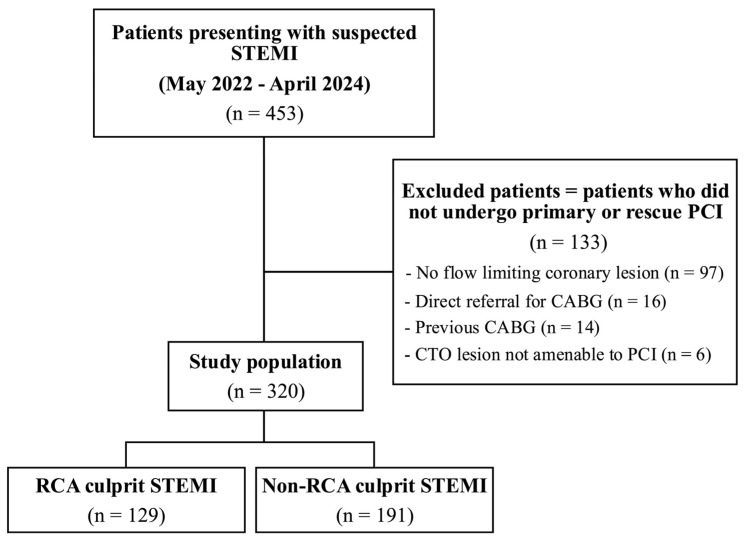
Study flow diagram. Abbreviations: CABG, coronary artery bypass grafting; CTO, chronic total occlusion; PCI, percutaneous coronary intervention; RCA, right coronary artery; STEMI, ST-elevation myocardial infarction.

The hospital’s cardiology procedural database (HealthTrack Medical Systems, Brisbane, Australia) was interrogated for all people presenting with acute STEMI due to coronary artery disease between May 2022 and April 2024. All patients undergoing native vessel primary PCI during their index angiogram were included. Exclusion criteria were no or non-obstructive coronary disease, spontaneous coronary artery dissection, up-front coronary artery bypass grafting (CABG) without initial PCI, suspected culprit lesions left for medical management, and culprit vessel PCI to a pre-existing bypass graft (Figure 1). The study population was then divided into two groups: those with an RCA culprit lesion and those with a non-RCA culprit lesion.

### 2.2. Clinical and Echocardiographic Variables

Baseline clinical characteristics were obtained from the procedural database and hospital electronic medical records. High-sensitivity troponin T was used as the cardiac biomarker assay at our institution (Roche Diagnostics Elecsys 5th-generation hs-cTnT assay), with an upper reference limit of <14 ng/L. Inpatient transthoracic echocardiogram (TTE) data were extracted, including left ventricular ejection fraction (LVEF) and specific measures of RV systolic function, namely, TAPSE and RV tissue doppler lateral annular systolic velocity (RV S′). As these measures of RV systolic function were not uniformly available for all patients, a combined variable of RV longitudinal dysfunction was chosen, defined as TAPSE < 17 mm or RV S′ < 10 cm/s according to contemporary published definitions [12]. Echocardiographic parameters were derived from the first available transthoracic echocardiogram performed during the index admission, which occurred within 48 h of presentation in 86% of the study cohort.

### 2.3. Coronary Angiographic Data

Coronary angiogram images were reviewed by experienced cardiologists. Standard criteria were used to define coronary artery dominance, as well as lesion location within the culprit vessel [13]. Severe angiographic stenosis was defined as ≥70%. The Thrombolysis in Myocardial Infarction (TIMI) flow grading system was used to assess antegrade perfusion in the culprit vessel [14]. Angiographically successful PCI was defined as stent deployment at the target lesion with residual stenosis < 20% [15]. In the RCA culprit STEMI cohort, the TIMI flow grading system was used to assess the patency of the RV marginal branch before and after PCI. The RV marginal branch was defined angiographically as the largest branch arising from the RCA along the acute margin of the heart, coursing towards the right ventricular wall [16]. In cases where multiple, similar-sized RV marginal branches were present, the branch demonstrating the lowest TIMI flow grade was used for analysis. The pre-PCI RV marginal branch TIMI flow was assessed on the baseline diagnostic angiogram acquisition. If the main RCA vessel was occluded proximally with TIMI 0 flow, the pre-PCI RV marginal branch TIMI flow was also graded TIMI 0. All angiographic assessments were performed blinded to clinical outcomes. Intra-observer reproducibility of pre- and post-PCI TIMI flow grading was assessed in a randomly selected subset of 20 patients, demonstrating excellent agreement (weighted kappa = 0.98 and 0.94, respectively). Procedural data were also recorded, including procedural access site, stent length and diameter, complications, and use of intraprocedural inotropic support and adjunct anti-thrombotic therapies (e.g., glycoprotein IIb/IIIa inhibitor).

### 2.4. Clinical Outcomes

Clinical outcome data were obtained from the medical records for the index admission, at 30 days and at 12 months, and included mortality, cardiogenic shock, new heart failure requiring diuresis, intensive care unit (ICU) admission, length-of-stay, and need for permanent pacemaker implantation. Cardiogenic shock was defined as sustained hypotension requiring initiation of inotropic or vasopressor medications and/or mechanical circulatory support. The primary endpoint was a composite of 30-day all-cause mortality and cardiogenic shock. This was selected for its objectivity and to align with those used in previous studies of similar patient populations [8,9,11]. Secondary outcome variables chosen for analysis were based on the existing literature [4,5,6,7,8,9,10,11,17,18].

### 2.5. Statistical Analysis

Data were analysed using STATA 18 (Stata Corp, College Station, TX, USA). Normality was tested with the Shapiro–Wilk test. Baseline characteristics were summarised using counts and percentages for categorical variables, means with standard deviations for normally distributed variables, and medians with interquartile ranges (IQRs) for non-normally distributed data. Comparisons between RCA and non-RCA-STEMI cohorts were made using chi-square tests for categorical variables and *t*-tests or Wilcoxon rank-sum tests for normal or non-normal continuous variables, respectively. To estimate the relative risks of the primary outcome and secondary outcomes, Poisson regression with robust standard errors was used to create risk ratios (RRs) and 95% confidence intervals (CIs). Continuous predictors were modelled as linear terms, and risk ratios were estimated per one-unit increase in the predictor variable, unless otherwise specified. Variables with *p* < 0.05 were considered for inclusion in multivariate analysis. Multivariate regression on RCA-STEMI patients was then performed using significant variables in univariate analysis to generate adjusted risk ratios. A two-sided *p*-value of <0.05 was considered significant.

## 3. Results

### 3.1. Characteristics of Study Cohort

A total of 453 individuals undergoing coronary angiography for suspected STEMI were identified. Of these, 320 underwent PCI to a culprit native vessel lesion and were eligible for inclusion (Figure 1). Among the 133 individuals excluded from analysis, the most common reason was the absence of a flow-limiting coronary lesion requiring intervention. Three quarters of the included patients were male (n = 236, 74%), and the median age was 62 years. A total of 129 (40%) had a culprit RCA lesion. Baseline clinical characteristics were similar between those with RCA and non-RCA culprit disease, although the RCA-STEMI cohort demonstrated a lower median troponin peak compared with the non-RCA-STEMI cohort (2876 ng/L [IQR: 1147–5163] vs. 4903 ng/L [IQR: 1503–10,728], *p* < 0.01), as well as a smaller proportion of patients without standard modifiable cardiovascular risk factors (SMuRF-less), namely, hypertension, hypercholesterolaemia, diabetes, or active smoking (10% vs. 20%, *p* = 0.03) (Table 1). Regarding angiographic and procedural characteristics, the RCA-STEMI group had shorter door-to-balloon time compared with the non-RCA-STEMI group (49 min [IQR: 32–73]) vs. 64 min [IQR: 41–96], *p* < 0.01) and a lower number of proximal vessel occlusions (28% vs. 41%, *p* = 0.01). The RCA-STEMI group had a marginally higher median total stent length than the non-RCA STEMI group (26 mm [IQR: 20, 38] vs. 24 mm [+18, 33], *p* = 0.01). There was a much lower incidence of LVEF < 40% in the RCA-STEMI cohort (2% vs. 28%, *p* < 0.01) but a higher incidence of patients with bystander coronary disease requiring subsequent revascularisation with CABG (9% vs. 3%, *p* = 0.02) (Table 2).

### 3.2. Clinical Outcomes

Primary and secondary outcomes are summarised for the RCA and non-RCA-STEMI cohorts in Table 3. The occurrence of the primary composite outcome was similar between the two groups: 12% in the RCA-STEMI group and 15% in the non-RCA-STEMI group; RR: 0.79 [95% CI: 0.44, 1.43], *p* = 0.44. All-cause mortality at 30 days was lower in the RCA-STEMI cohort (2% vs. 8%, RR: 0.19 [95% CI: 0.04, 0.79], *p* = 0.01), and a similar trend was observed at 12 months. The incidence of cardiogenic shock was similar between both groups, occurring in 15 patients with RCA-STEMI and 24 patients with non-RCA culprit lesions (12% vs. 13%, RR: 0.93 [95% CI: 0.51, 1.69], *p* = 0.80). The number of individuals affected by inpatient heart failure requiring diuresis was numerically higher for the non-RCA-STEMI cohort without reaching statistical significance (4% vs. 9%, RR: 0.43 [95% CI: 0.16, 1.14], *p* = 0.08). There were no significant differences for the other secondary outcomes of length of hospital stay, permanent pacemaker requirement at index admission or 12 months, or 12-month readmission for heart failure or acute coronary syndrome.

### 3.3. Predictors of the Composite Primary Outcome

Table 4 summarises the results of the univariate analysis for clinical, echocardiographic and angiographic factors and their association with the composite 30-day primary outcome in patients with RCA or non-RCA-STEMI. In both groups, cardiac arrest on presentation (RR: 5.04 [95% CI: 3.03, 8.38], *p* < 0.01) and peak troponin level (per 1000 units RR: 1.04 [95% CI: 1.03, 1.05], *p* < 0.01) were independently associated with a worse primary outcome.

#### 3.3.1. Echocardiographic Associations with Primary Outcome

Echocardiographic assessment of RV longitudinal function was incomplete (TAPSE: 25% missing; RV S′: 21% missing). Patients without available RV measurements had higher rates of cardiac arrest at presentation and worse clinical outcomes (Supplementary Table S1). In univariate and multivariate analyses of those with available data for RV function, the presence of RV longitudinal dysfunction was predictive of the composite primary outcome in the RCA-STEMI group (multivariate: RR: 2.90 [95% CI: 1.01, 8.27], *p* = 0.047) (Table 5). This association was more prominent for impaired RV S′ (RR: 5.88 [95% CI: 2.38, 14.51], *p* < 0.01) than reduced TAPSE (RR: 2.30 [95% CI: 0.87, 6.12], *p* = 0.09). The presence of LVEF < 40% was associated with higher rates of the composite primary endpoint in the non-RCA-STEMI cohort (RR: 4.98 [95% CI: 1.99, 12.43], *p* < 0.01) but did not reach significance in the RCA-STEMI group (RR: 3.05 [95% CI: 0.56, 16.49], *p* = 0.20), although there were very few observed events.

#### 3.3.2. Procedural and Angiographic Associations with Primary Outcome

In both groups, radial artery access was associated with a lower event rate and intraprocedural use of glycoprotein IIb/IIIa inhibitor with a higher rate of the primary outcome (Table 4). Severe two-vessel coronary disease was present in 86 patients (26%), and severe three-vessel disease was present in 42 patients (13%). The presence of severe multivessel disease was not associated with the composite primary outcome in either infarct territory subgroup. Among those with multivessel disease, 67 patients (52%) underwent revascularisation of bystander lesions during the index admission, either via staged PCI or CABG. Complete revascularisation in these patients was associated with a strongly reduced rate of the composite primary outcome in the non-RCA-STEMI subgroup (RR: 0.09 [95% CI: 0.01–0.67], *p* < 0.01) but not in those presenting with RCA-STEMI (RR: 0.78 [95% CI: 0.19–3.18], *p* = 0.73). Notably, proximal segment culprit site did not meet significance for either group (RCA-STEMI RR: 1.29 [95% CI: 0.47, 3.53], *p* = 0.61; non-RCA-STEMI RR: 1.89 [95% CI: 0.95, 3.78], *p* = 0.07). In contrast, for patients with RCA-STEMI, impaired coronary flow in the RV marginal branch (TIMI grade < 3) was associated with worse 30-day outcomes on univariate analysis, both when it was present before PCI (RR: 4.53 [95% CI: 1.06, 19.37], *p* = 0.04) and after (RR: 7.21 [95% CI: 2.67, 19.46], *p* < 0.01). This association was also significant for reduced RV marginal flow post-PCI on multivariate analysis (RR: 4.19 [95% CI: 1.10, 15.94], *p* = 0.035) (Table 5). Finally, individuals who had TIMI flow grade < 3 in their RV marginal branch after PCI had higher observed rates of ICU admission for cardiogenic shock (RR: 12.63 [95% CI: 2.76, 57.76], *p* < 0.01) and new heart failure requiring inpatient diuretic therapy (RR: 14.43 [95% CI: 1.65, 125.03], *p* = 0.02). No instances of intentional RV marginal branch wiring or balloon angioplasty were performed during the index PCI.

## 4. Discussion

This single-centre retrospective analysis of contemporary STEMI outcomes demonstrates an association between impaired RV marginal branch perfusion on post-PCI angiography, RV longitudinal dysfunction on echocardiography, and worse clinical outcomes among patients with RCA-STEMI. Within the RCA-STEMI cohort, individuals with TIMI flow < 3 in the RV marginal branch post-PCI were 4-fold more likely to sustain the composite 30-day primary outcome of all-cause mortality or cardiogenic shock compared to those with TIMI 3 flow. This builds on a prior study from 2010 which showed that RCA occlusions proximal to the RV marginal branch were similarly accompanied by higher rates of cardiogenic shock and 30-day mortality [8]. However, a more recent study by Hamaguchi et al. [19] reported no significant difference in the primary composite major adverse cardiovascular event (MACE) endpoint between proximal and non-proximal RCA occlusions in RCA culprit STEMI, suggesting that outcomes may not be driven by the site of main vessel occlusion. Assali et al. demonstrated that in the early era of PCI (2001-5), the presence of TIMI grade 3 flow in the RV marginal branch was associated with improved 30-day survival in individuals presenting with inferior STEMI [6]. Despite this early insight, there has been a paucity of more recent evidence to inform the significance of RV marginal branch patency in STEMI presentations. Our study provides contemporary observational data on the association between impaired RV marginal flow after PCI and adverse short-term outcomes. While this knowledge may help early risk stratification, it remains unclear whether outcomes can be improved by selectively intervening on the RV marginal branch to improve flow after PCI to the main RCA vessel. Current practice for RV marginal branch protection (e.g., branch vessel wiring or balloon angioplasty) is guided by anatomical judgement and proceduralist preference. Notably, there were no instances of RV marginal branch wiring within our cohort, reflecting contemporary operator preference to prioritise main vessel reperfusion.

Our results also support an association between RV longitudinal dysfunction and higher 30-day mortality and cardiogenic shock in patients with RCA-STEMI. This complements and expands on emerging evidence supporting the prognostic value of echocardiographic markers of RV dysfunction in patients with STEMI. Previous observational studies of STEMI complicated by cardiogenic shock found that TAPSE < 14 mm [9] and RV S′ < 10.5 cm/s [17] were independently associated with increased all-cause mortality. Similarly, in line with the current data, other reports focusing on RCA culprit STEMI demonstrated that impaired RV free-wall global longitudinal strain was accompanied by higher 30-day mortality [10,18].

As catheter-based temporary RV mechanical circulatory support systems become more widely available, early identification of patients at risk of haemodynamic deterioration is becoming increasingly important. The use of newer advanced haemodynamic platforms requires an intimate understanding of cardiac physiology and the alterations that can occur due to STEMI to facilitate early identification of people who may benefit from their use. The Impella-RP (Abiomed, Danvers, USA) is a percutaneous RV assist device shown to be effective in improving haemodynamics in patients with cardiogenic shock and severe RV dysfunction [20]. More recently, it has also been shown to benefit management of RV failure after cardiac surgery [21]. Previous observational data have suggested improved outcomes with early device deployment in the management of cardiogenic shock [22], underscoring the value of timely risk stratification. Our findings identify angiographic and echocardiographic variables associated with adverse outcomes in RCA-STEMI; whether these markers can be used to guide selection for therapies such as RV mechanical circulatory support remains uncertain and requires prospective evaluation.

Several findings merit further consideration. RCA-STEMI was not associated with differences in the composite primary endpoint but appeared to be associated with lower 30-day mortality. Although RCA-STEMI patients had shorter door-to-balloon times and fewer proximal culprit lesions, rates of cardiogenic shock were similar to those observed in non-RCA STEMI. This suggests that haemodynamic compromise is influenced by factors beyond epicardial lesion location and reperfusion time alone, including right ventricular involvement and baseline clinical status. Notably, door-to-balloon time and proximal vessel location were not independent predictors of the primary outcome in our cohort. The lack of difference in inpatient permanent pacemaker implantation between infarct territories likely reflects the very low event rate (n = 3), limiting statistical power. The observed association between treatment strategies (glycoprotein IIb/IIIa inhibitor use and vascular access site) and outcomes was likely influenced by confounding by indication. Such findings likely reflect underlying procedural complexity and haemodynamic instability rather than independent causal effects. An unexpected observation was that post-PCI culprit vessel TIMI flow < 3 was not associated with adverse outcomes in non-RCA STEMI. We speculate that in anterior infarction, a larger infarct size and resultant left ventricular dysfunction may dominate prognosis, potentially attenuating the discriminatory value of TIMI flow grade. Alternatively, event rates within the non-RCA subgroup with post-PCI TIMI < 3 may have been insufficient to detect a significant association in our study. Finally, the association between reduced RV marginal branch flow and increased diuretic requirement may reflect the dynamic haemodynamic course of RV dysfunction, where early fluid resuscitation may later give way to volume overload and the need for diuresis.

This study has limitations owing largely to its retrospective, single-centre and observational nature. In addition, we obtained wide confidence intervals around several point estimates, which reflects low event rates and limited statistical power. Regarding outcomes, the definition of cardiogenic shock was broad: sustained hypotension requiring initiation of inotropic or vasopressor therapy and/or mechanical circulatory support. This was based on the haemodynamic and clinical variables available within the dataset. Objective markers of end-organ hypoperfusion were not consistently recorded and therefore could not be incorporated into the definition. This broader definition may have introduced heterogeneity within the cardiogenic shock cohort and the potential for misclassification. Our definition also did not include normotensive shock. Regarding angiographic assessments, TIMI flow grading in a side branch is inherently subjective, and although blinded assessment and reproducibility testing were performed, misclassification cannot be entirely excluded. Furthermore, we did not systematically adjudicate the mechanism of reduced TIMI flow (e.g., no-reflow, distal embolisation, compromise after stent implantation, or more global haemodynamic impairment), which may have distinct pathophysiological implications, and this represents a limitation of the study. Our analysis of patients without available results for RV longitudinal function showed that they had higher rates of cardiac arrest at presentation and worse clinical outcomes, suggesting that missingness was not random and may reflect greater haemodynamic instability. The results should therefore be interpreted with caution. In addition, TAPSE and RV S′ are load-dependent indices of RV function and may be influenced by haemodynamic status and the use of vasoactive agents, mechanical ventilation, or mechanical circulatory support. Echocardiography timing was not standardised, and measurements were not routinely obtained during or immediately following PCI. These factors may have introduced variability in RV functional assessment and should be considered when interpreting the results. Due to limitations in data capture within the hospital electronic medical records system, 30-day and 12-month outcome data may have been incomplete for some patients, particularly those who were transferred from interstate, private-sector hospitals or were international citizens. This loss of follow-up may have led to an under-estimation of adverse events, potentially hindering identification of the long-term clinical predictors. Given the observational design, the findings of this study should be considered hypothesis-generating and not used to guide therapeutic decision making without prospective validation.

## 5. Conclusions

In this retrospective analysis, impaired flow in the RV marginal branch post-PCI and echocardiographic features of RV longitudinal dysfunction were independently associated with cardiogenic shock and 30-day all-cause mortality in people presenting with RCA culprit vessel STEMI. This sheds light on the importance of RV involvement in this subgroup of patients and the prognostic relevance of RV marginal branch involvement, which it is often left to the operator’s discretion to intervene upon with no clear direction from current clinical guidelines. Future prospective studies are now required to evaluate whether specific interventions to restore RV marginal branch patency will improve outcomes and whether the implementation of echocardiographic criteria of RV dysfunction can identify a group that may benefit from emerging RV mechanical support therapies.

## Figures and Tables

**Table 1:** Baseline clinical characteristics in RCA-STEMI and non-RCA-STEMI cohorts.

Baseline Characteristic	Total Cohort n = 320	RCA-STEMI n = 129	Non-RCA-STEMI n = 191	*p*-Value
Age, median [IQR]	62 [54, 73]	60 [54, 72]	64 [54, 74]	0.44
Male sex, n (%)	236 (74)	90 (70)	146 (76)	0.18
Prior ischaemic heart disease, n (%)	54 (17)	29 (22)	34 (18)	0.59
Prior atrial fibrillation, n (%)	14 (4)	3 (2)	11 (6)	0.14
Prior heart failure, n (%)	10 (3)	6 (5)	4 (2)	0.20
- HFpEF	1 (0.3)	0	1 (0.5)	0.40
- HFrEF	8 (3)	5 (4)	3 (2)	0.20
Active smoking, n (%)	108 (34)	47 (36)	61 (32)	0.38
Diabetes, n (%)	76 (24)	29 (22)	47 (25)	0.66
Hypertension, n (%)	137 (43)	55 (43)	82 (43)	0.96
Hypercholesterolaemia, n (%)	103 (32)	43 (33)	60 (31)	0.72
SMuRF-less patients, n (%)	51 (16)	13 (10)	38 (20)	0.03
Peripheral vascular disease, n (%)	5 (2)	1 (0.7)	4 (2)	0.35
COAD, n (%)	16 (5)	5 (4)	11 (6)	0.44
Cardiac arrest on presentation, n (%)	40 (13)	13 (10)	27 (14)	0.28
Thrombolysis on presentation, n (%)	41 (13)	13 (10)	28 (15)	0.23
Troponin peak (ng/L), median [IQR]	3906 [1248, 8089]	2876 [1147, 5163]	4903 [1503, 10,728]	<0.01

Abbreviations: COAD, Chronic Obstructive Airways Disease. HFpEF, Heart Failure with Preserved Ejection Fraction. HFpEF was defined as a pre-existing diagnosis of heart failure with an ejection fraction of ≥50%. HFrEF, Heart Failure with Reduced Ejection Fraction. HFrEF was defined as a pre-existing diagnosis of heart failure with an ejection fraction of <40%. IQR, interquartile range. RCA, right coronary artery. SMuRF-less patients were defined as having none of the standard modifiable cardiovascular risk factors of hypertension, hypercholesterolaemia, diabetes, or active smoking.

**Table 2:** Baseline angiographic and echocardiographic characteristics in RCA-STEMI and non-RCA-STEMI cohorts.

Baseline Characteristic	Total Cohort n = 320	RCA-STEMI n = 129	Non-RCA-STEMI n = 191	*p*-Value
Angiographic/Procedural				
Door-to-balloon time, median [IQR]	56 [37, 86]	49 [32, 73]	64 [41, 96]	<0.01
Radial access, n (%)	288 (90)	115 (89)	173 (91)	0.68
Intraprocedural glycoprotein IIb/IIIa inhibitor, n (%)	56 (18)	24 (19)	32 (17)	0.67
Culprit vessel TIMI < 3 flow post-PCI, n (%)	55 (17)	26 (20)	29 (15)	0.24
RV marginal branch TIMI < 3 flow pre-PCI, n (%) *	-	76 (59)	-	N/A
RV marginal branch TIMI < 3 flow post-PCI, n (%) *	-	28 (22)	-	N/A
Number of stents, median [IQR]	1 [1, 1]	1 [1, 1]	1 [1, 1]	0.64
Stent diameter, median [IQR]	3.0 [2.8, 3.5]	3.5 [3.0, 3.5]	3.0 [2.8, 3.5]	<0.01
Total stent length, median [IQR]	26 [18, 35]	26 [20, 38]	24 [18, 33]	0.01
Proximal vessel occlusion, n (%)	115 (36)	36 (28)	79 (41)	0.01
Severe two-vessel disease, n (%)	86 (27)	37 (29)	49 (26)	0.55
Severe three-vessel disease, n (%)	42 (13)	20 (16)	22 (12)	0.30
LMCA culprit or bystander disease, n (%)	10 (3)	3 (2)	7 (4)	0.50
Revascularisation of bystander disease with PCI, n (%)	54 (17)	27 (21)	27 (14)	0.11
Revascularisation of bystander disease with CABG, n (%)	16 (5)	11 (9)	5 (3)	0.02
Echocardiographic				
LV ejection fraction, median [IQR]	48 [41, 55]	53 [48, 57]	44 [36, 50]	<0.01
LV ejection fraction < 40%, n (%)	57 (18)	3 (2)	54 (28)	<0.01
TAPSE < 17 mm, n (%) **	48 (20)	23 (23)	25 (18)	0.24
RV S′ < 10 cm/s, n (%) ***	42 (17)	21 (19)	21 (15)	0.17
RV longitudinal dysfunction, n (%)	71 (22)	35 (27)	36 (19)	0.08

Abbreviations: CABG, coronary artery bypass grafting; IQR, interquartile range; LMCA, left main coronary artery; LV, left ventricle; PCI, percutaneous coronary intervention; RV longitudinal dysfunction was defined as the presence of TAPSE < 17 mm or RV S′ < 10 cm/s; RCA, right coronary artery; RV, right ventricle; RV S′, right ventricular tissue doppler lateral annular systolic velocity; severe two-vessel disease was defined as the presence of significant stenosis (≥70%) in two major epicardial coronary arteries; severe three-vessel disease was defined as the presence of significant stenosis (≥70%) in three major epicardial coronary arteries; TAPSE, tricuspid annular plane systolic excursion; TIMI, Thrombolysis in Myocardial Infarction flow grading system. * Parameters only investigated in the RCA-STEMI cohort; ** 80 patients did not have TAPSE assessed on inpatient echocardiography; *** 67 patients did not have RV S′ assessed on inpatient echocardiography. Corresponding percentages shown for these parameters were derived only from those patients for whom the data were available.

**Table 3:** Primary and secondary outcome events.

Outcome Variable	Total Cohort n = 320	RCA-STEMI n = 129	Non-RCA-STEMI n = 191	Risk Ratio [95% CI]	*p*-Value
Primary Outcome					
Composite primary, n (%)	43 (13)	15 (12)	28 (15)	0.79 [0.44, 1.43]	0.44
Cardiogenic shock, n (%)	39 (12)	15 (12)	24 (13)	0.93 [0.51, 1.69]	0.80
30-day mortality, n (%)	18 (6)	2 (2)	16 (8)	0.19 [0.04, 0.79]	0.01
Secondary Outcomes					
Length of stay (days), median [IQR]	4 [3, 5]	3 [3, 5]	4 [3, 5]	1.09 [0.86, 1.40]	0.46
Length of stay > 4 days, n (%)	86 (27)	35 (27)	51 (27)	1.01 [0.70, 1.47]	0.93
Inpatient heart failure, n (%)	22 (7)	5 (4)	17 (9)	0.43 [0.16, 1.14]	0.08
ICU admission for cardiogenic shock, n (%)	29 (9)	9 (7)	20 (10)	0.67 [0.31, 1.42]	0.29
Inpatient PPM implantation, n (%)	3 (1)	1 (1)	2 (1)	0.72 [0.07, 7.91]	0.79
12-month mortality, n (%)	29 (9)	6 (5)	23 (12)	0.39 [0.16, 0.92]	0.02
12-month heart failure admission, n (%)	17 (5)	6 (5)	11 (6)	0.81 [0.31, 2.13]	0.67
12-month ACS re-admission, n (%)	11 (3)	5 (4)	6 (3)	1.23 [0.39, 3.96]	0.72
12-month PPM implantation, n (%)	8 (2)	2 (2)	6 (3)	0.49 [0.10, 2.41]	0.37

Abbreviations: ACS, acute coronary syndrome; cardiogenic shock was defined as sustained hypotension requiring vasopressor support and/or mechanical circulatory support; CI, confidence interval; ICU, intensive care unit; inpatient heart failure was defined as documented heart failure requiring inpatient diuretic therapy; PPM, permanent pacemaker; RCA, right coronary artery.

**Table 4:** Clinical, echocardiographic, and angiographic predictors of the composite primary outcome in the RCA-STEMI and non-RCA-STEMI cohorts.

Predictor Variable	Total Cohort		RCA-STEMI Cohort		Non-RCA-STEMI Cohort	
	Risk Ratio [95% CI]	*p*-Value	Risk Ratio [95% CI]	*p*-Value	Risk Ratio [95% CI]	*p*-Value
Baseline/Presentation Characteristics						
Age (per 10 years)	1.09 [0.90, 1.31]	0.37	1.11 [0.77, 1.62]	0.57	1.08 [0.87, 1.33]	0.50
Male sex	0.92 [0.50, 1.71]	0.79	1.19 [0.40, 3.53]	0.75	0.77 [0.36, 1.63]	0.50
Thrombolysis on STEMI presentation	1.32 [0.63, 2.78]	0.46	2.23 [0.72, 6.92]	0.17	0.97 [0.36, 2.59]	0.95
Cardiac arrest on presentation	5.04 [3.03, 8.38]	<0.01	5.95 [2.51, 14.10]	<0.01	4.56 [2.43, 8.55]	<0.01
Troponin peak, per 1000 units	1.04 [1.03, 1.05]	<0.01	1.08 [1.04, 1.13]	<0.01	1.04 [1.03, 1.05]	<0.01
Severe two-vessel disease	1.66 [0.81, 3.41]	0.16	1.30 [0.41, 4.12]	0.66	1.96 [0.78, 4.95]	0.15
Severe three-vessel disease	0.84 [0.27, 2.62]	0.76	-	-	1.78 [0.56, 5.63]	0.33
Echocardiographic Characteristics						
LV ejection fraction < 40%	3.14 [1.68, 5.89]	<0.01	3.05 [0.56, 16.49]	0.20	4.98 [1.99, 12.43]	<0.01
TAPSE < 17 mm *	1.30 [0.64, 2.62]	0.47	2.30 [0.87, 6.12]	0.09	0.80 [0.26, 2.45]	0.69
RV S′ < 10 cm/s **	2.56 [1.43, 4.59]	<0.01	5.88 [2.38, 14.51	<0.01	1.35 [0.52, 3.52]	0.54
RV longitudinal dysfunction	1.88 [1.06, 3.32]	0.03	5.37 [1.97, 14.67]	<0.01	0.94 [0.38, 2.30]	0.89
Angiographic Characteristics						
Radial access	0.29 [0.16, 0.50]	<0.01	0.33 [0.12, 0.91]	0.03	0.26 [0.13, 0.50]	<0.01
Door-to-balloon time	1.01 [1.00, 1.03]	0.11	1.04 [0.99, 1.10]	0.14	1.01 [0.99, 1.03]	0.43
Intraprocedural glycoprotein IIb/IIIa inhibitor	3.39 [1.99, 5.79]	<0.01	2.92 [1.14, 7.44]	0.03	3.73 [1.95, 7.11	<0.01
Proximal vessel occlusion	1.70 [0.98, 2.96]	0.06	1.29 [0.47, 3.53]	0.61	1.89 [0.95, 3.78]	0.07
Culprit vessel TIMI < 3 flow post-PCI	1.90 [1.01, 3.90]	0.04	3.92 [1.50, 10.23]	<0.01	1.08 [0.36, 3.27]	0.88
RV marginal branch TIMI < 3 flow pre-PCI ***	-	-	4.53 [1.06, 19.37]	0.04	-	-
RV marginal branch TIMI < 3 flow post-PCI ***	-	-	7.21 [2.67, 19.46]	<0.01	-	-

Abbreviations: CI, confidence interval; LV, left ventricle; PCI, percutaneous coronary intervention; RV longitudinal dysfunction was defined as the presence of TAPSE < 17 mm or RV S′ < 10 cm/s; RCA, right coronary artery; RV, right ventricle; RV S′, right ventricular tissue doppler lateral annular systolic velocity; TAPSE, tricuspid annular plane systolic excursion; TIMI, Thrombolysis in Myocardial Infarction flow grading system; * 80 patients (25%) did not have TAPSE assessed on inpatient echocardiography; ** 67 patients (21%) did not have RV S′ assessed on inpatient echocardiography; *** parameters only investigated in the RCA-STEMI cohort.

**Table 5:** Multivariate predictors of the primary composite outcome in the RCA-STEMI cohort.

Predictor Variable	Risk Ratio [95% CI]	*p*-Value
Cardiac arrest on presentation	2.48 [0.70, 8.80]	0.161
Troponin peak, per 1000 units	1.01 [0.95, 1.08]	0.642
Culprit vessel TIMI < 3 flow post-PCI	1.76 [0.77, 4.03]	0.181
RV marginal branch TIMI < 3 flow post-PCI	4.19 [1.10, 15.94]	0.035
RV longitudinal dysfunction on echocardiogram	2.90 [1.01, 8.27]	0.047

Abbreviations: CI, confidence interval; PCI, percutaneous coronary intervention; RV, right ventricle; TIMI, Thrombolysis in Myocardial Infarction flow grade.

## Data Availability

The data underlying this article will be shared on reasonable request to the corresponding author.

## Supplementary Materials

The following supporting information can be downloaded at https://www.mdpi.com/article/10.3390/jcm15072595/s1: Table S1: Comparison of patients with and without any available data for right ventricular longitudinal function.

## Abbreviations

The following abbreviations are used in this manuscript:

STEMI	ST-elevation myocardial infarction
RCA	Right coronary artery
PCI	Percutaneous coronary intervention
RV	Right ventricle
TAPSE	Tricuspid annular plane systolic excursion
RV S′	RV tissue doppler lateral annular systolic velocity
TIMI	Thrombolysis in Myocardial Infarction
CABG	Coronary artery bypass grafting
TTE	Transthoracic echocardiogram
LVEF	Left ventricular ejection fraction
ICU	Intensive care unit
IQR	Interquartile range
LMCA	Left main coronary artery
RR	Risk ratio
CI	Confidence interval
