# Supplementary material for: Predictors of Adverse 30-Day Outcomes After Right Coronary Artery ST-Elevation Myocardial Infarction
Source: J Clin Med. 2026 Mar 28;15(7):2595. doi: 10.3390/jcm15072595 (PMC13073013; doi:10.3390/jcm15072595)
Supplement: Supplementary file 1 [file jcm-15-02595-s001.zip › jcm-4133269-supplementary.pdf]

Supplementary Table S1. Comparison of patients with and without any available data for right ventricular longitudinal function.

**Variable**	**RV data available (n=286)**	**No RV data available (n=34)**	**p-value**
Baseline characteristics			
Age, median [IQR]	62 [53, 73]	68 [57, 77]	0.072
Male sex, n (%)	213 (74.5)	23 (67.6)	0.392
Cardiac arrest on presentation, n (%)	32 (11.2)	8 (23.5)	0.040
Thrombolysis on presentation, n (%)	38 (13.3)	3 (8.8)	0.462
Troponin peak (ng/L), median [IQR]	3946 [1249, 8420]	2890 [785, 6615]	0.343
Outcome variables			
Composite primary outcome, n (%)	31 (10.8)	12 (35.3)	<0.001
12-month mortality, n (%)	15 (5.2)	14 (41.2)	<0.001

Abbreviations: RV, right ventricular; IQR, inter-quartile range; n, number,
